# Conceptualization of the latent structure of autism: further evidence and discussion of dimensional and hybrid models

**DOI:** 10.1007/s00787-022-02062-y

**Published:** 2022-08-25

**Authors:** Sarah Wittkopf, Anika Langmann, Veit Roessner, Stefan Roepke, Luise Poustka, Igor Nenadić, Sanna Stroth, Inge Kamp-Becker

**Affiliations:** 1 Department of Child and Adolescent Psychiatry, Psychosomatics and Psychotherapy, Medical Clinic, Philipps-University, Marburg, Germany; 2 Department of Child and Adolescent Psychiatry, Technical University Dresden, Dresden, Germany; 3 Department of Psychiatry, Charité Universitätsmedizin Berlin, Campus Benjamin Franklin, Berlin, Germany; 4 Department of Child and Adolescent Psychiatry and Psychotherapy, University Medical Center Göttingen, Göttingen, Germany; 5 Department of Psychiatry and Psychotherapy, Medical Clinic, Philipps-University Marburg, Marburg, Germany

**Keywords:** Autism spectrum disorder, Autism classification, Latent structure hybrid model, Factor mixture modeling

## Abstract

Autism spectrum disorder (ASD) might be conceptualized as an essentially dimensional, categorical, or hybrid model. Yet, current empirical studies are inconclusive and the latent structure of ASD has explicitly been examined only in a few studies. The aim of our study was to identify and discuss the latent model structure of behavioral symptoms related to ASD and to address the question of whether categories and/or dimensions best represent ASD symptoms. We included data of 2920 participants (1–72 years of age), evaluated with the Autism Diagnostic Observation Schedule (Modules 1–4). We applied latent class analysis, confirmatory factor analysis, and factor mixture modeling and evaluated the model fit by a combination of criteria. Based on the model selection criteria, the model fits, the interpretability as well as the clinical utility we conclude that the hybrid model serves best for conceptualization and assessment of ASD symptoms. It is both grounded in empirical evidence and in clinical usefulness, is in line with the current classification system (DSM-5) and has the potential of being more specific than the dimensional approach (decreasing false positive diagnoses).

**Supplementary Information:**

The online version contains supplementary material available at 10.1007/s00787-022-02062-y.

## Introduction

There has been a long-lasting and unresolved debate whether mental disorders are best conceptualized by a continuum of severity in one or more dimensions or as discrete categories of distinct disorders. Similarly, the concept of autism spectrum disorder (ASD) has shifted from a strictly defined “childhood condition” usually associated with profound deficits, challenges in language and intellectual functioning, to a wider concept including individuals with mild symptoms or autistic traits and to a lifelong condition including individuals who are not diagnosed until adulthood [1]. Associated with this conceptual shift, there is considerable heterogeneity across the ASD phenotype concerning the expression and severity of symptoms, adaptive functions, cognitive and speech skills as well as co-occurring conditions [1]. Research on diagnostic criteria in ASD has resulted in several adaptations emerging different conceptualizations of ASD [2]. In an ongoing debate, ASD is either conceptualized as essentially dimensional (i.e., symptoms are continuously distributed across the general population) [3–5], categorical (i.e., dichotomous delineation between affected and unaffected individuals) [6] or a condition that combines categorical and dimensional attributes, the so-called hybrid model [7–11]. In previous classification systems DSM-IV [12] and ICD-10 [13], ASD was conceptualized as a multi-categorical disorder with different subtypes. However, growing evidence has shown that these subtypes cannot be differentiated as distinct, empirically defined subgroups according to the specified criteria [14]. Consequently, the authors of the DSM-5 conceptualized a hybrid approach for ASD: Dimensional individual differences in symptom severity and general impairment are considered within a categorical umbrella term of ASD [11, 15, 16].

The conceptualization of ASD symptoms as dimensional or categorical has immediate implications for the design, analysis and interpretation of (biological) studies on assessment, classification and treatment of ASD [11] and is thus of great importance to both, the clinical and neurobiological understanding of ASD. The *categorical* model makes a dichotomous distinction between diagnostic groups (i.e., condition is absent or present) indicating a clear diagnostic decision. However, due to the fact that ASD shares symptoms with other disorders and is accompanied by several comorbidities, this decision is quite difficult in many cases. In research contexts, case–control-designs (ASD versus Non-ASD) are applied, revealing group differences in, for example, brain structure and function [17]. If a *dimensional* model is assumed, the presence of symptoms is continuously distributed across the general population and severity thresholds are needed in order to make a diagnostic decision. This conceptualization is in line with the results of studies indicating that signs of ASD occur frequently in the general population [18]. In clinical practice, the nature of the diagnostic threshold is of great importance but thresholds are necessarily arbitrary and/or do not reach reliability among clinicians [19]. In research designs, ASD symptoms are measured as quantitative traits and regression or latent factor approaches are appropriate. Graded alterations in, e.g., brain development and overlapping typical brain trajectories can be postulated. The *hybrid* model [20] assumes qualitative differences between individuals with and without ASD (categorical aspect), and, at the same time, dimensional heterogeneity concerning the intensity of one or more-dimensional constructs within the ASD and non-ASD groups. For clinical purposes, accurate characterization of different constructs (e.g., social communication and repetitive, stereotyped behaviors) is required to decide on the presence versus absence of ASD. In research contexts, hybrid models examine whether ASD symptoms measure one or more dimensions as well as whether individual differences in those dimensions result from one or more groups of individuals [11]. A further important line of research, beyond the scope of the current work, addresses the latent structure of ASD investigating whether the symptoms of ASD itself should be conceptualized as a unitary or multidimensional construct (see [21]).

To our knowledge, the conceptualization of the distribution of ASD symptoms as dimensional or not has explicitly been examined in only a few studies and results are contradictory. While Frazier et al. [11] and Georgiades et al. [22] found evidence for the hybrid model, Kim et al. [5] and Uljarevic et al. [23] described ASD symptoms as being dimensional. A key design element in evaluating the latent (taxonomic) structure is the selection of assessment instruments and samples. In all studies so far, parent report (screening) measures were used. However, said measures may be vulnerable to several biases (recall- or confirmation-bias, halo-, contrast- or expectancy-effects, social desirability, etc.), which could likely impact results. Additional studies are needed, incorporating different types of symptom assessments, such as behavior observations by trained examiners, to inform the debate on the taxonomic nature of the ASD psychopathology. Another key aspect concerns the composition of the sample as it is important to assess the full range of heterogeneity and severity of ASD symptoms in ASD as well as in other mental disorders. In previous research, mostly healthy controls, siblings of children with ASD or their parents were examined as control groups. However, another highly relevant sample includes individuals with other mental disorders showing some ASD symptoms. The aim of our study was to further enrich the existing debate about the latent structure of ASD with findings on observed behavioral data. Therefore, we analyzed data of a large, clinically referred and well characterized sample of individuals with ASD and a group of individuals with symptom overlap with ASD but other mental disorders or no clinical diagnosis. Based on behavior observations in a large clinical sample, this study aims to model the latent structure of ASD symptoms as dimensional, categorical or hybrid and to discuss the clinical implications of each model.

## Method

### Participants

For analyses, a subsample which only contained complete datasets was extracted from ASD-Net, a research network including a large clinical database [24]. For all subjects, a diagnosis of ASD or non-ASD based on “gold standard” best-estimate clinical diagnosis (BEC) was available. BEC diagnoses rely on the evaluation of two trained and experienced clinicians following extensive examination and review of all available information (IQ, neuropsychological testing, reports from other institutions, home videos, standardized examination, differential diagnostic examination with established structured questionnaires, structural clinical interviews) to arrive at a clinical consensus diagnostic decision. Diagnoses were based on ICD-10 [13]. The procedure was approved by the local ethics committee (AZ: 92/20). Due to the retrospective nature of data collection and analysis based on anonymized data, the need for informed consent was waived by the ethics committee.

The non-ASD group consisted of individuals with other mental (e.g., anxiety, mood, attention deficit and hyperactivity or personality disorder), or no axis one diagnoses (11.4% of the sample), but other developmental disorders or delays (see table S1, available online). Comorbid disorders were no exclusion criterion. The study included data of 2920 participants (1–72 years of age, 19.0% female). Table 1 shows further descriptive data on age, IQ and IQ level.

**Table 1:** Sample characteristics

	*n*	*Age Mean (SD)*	*n*	*IQ Mean (SD)*	*n*	*IQ-level Mean (SD)*
Module 1	438	5.52 (3.92)	61	70.00 (20.00)	187	5.07 (1.05)
Module 2	555	6.31 (3.34)	264	80.69 (19.31)	363	3.91 (1.02)
Module 3	1003	10.00 (2.60)	820	98.96 (17.73)	886	3.01 (0.83)
Module 4	924	26.83 (11.71)	792	104.42 (15.81)	843	2.89 (0.73)

IQ-level: 1 =  > 129; 2 = 115–129; 3 = 85–114; 4 = 70–84; 5 = 50–69; 6 = 35–49

*n* Sample size

### Measures

The Autism Diagnostic Observation Schedule (ADOS-G and ADOS-2) [25–29] was administered to all participants in order to assess ASD symptoms and severity. The ADOS is a semi-structured and standardized observation tool that consists of four modules to be administered on the basis of the individual’s level of expressive language and chronological age. Substantial interrater and test–retest reliability for individual items, excellent interrater reliability within domains and excellent internal consistency was found for the ADOS [29]. Data for this study were checked for inappropriate administrations along the ADOS manual criteria and deviating datasets were excluded. Coding indicates increasing symptom severity (0, 1, 2 and 3). Behavioral items which are part of each modules’ diagnostic algorithm were used for analyses and grouped into two domains: Social Affect (SA) and Restricted and Repetitive Behaviors (RRB). SA and RRB scores represent expected a posteriori factor scores [30]. 438 participants (58.7% with a diagnosis of ASD) were tested with Module 1, 555 participants received Module 2 (39.5% ASD), whereas 1003 individuals were observed with Module 3 (44.3% ASD) and  924 with Module 4 (54.4% ASD).

### Analyses

Following ADOS manual conventions, item codes were calculated. Analyses were conducted in MPlus (version 8.4) [30]. To avoid circularity, data-based analyses were carried out without considering assigned diagnoses. Model selection was conducted along the lines of previously published recommendations [31], relying on the Bayesian information criteria (BIC) [32], the Akaike information criteria (AIC) [33], inspection of factor value distributions (via density plots [34] and Q–Q plots) to judge whether factor scores were normally distributed or not, and item plots (i.e., interpretability). The following analyses were carried out separately for each of the ADOS modules, because the dimensions SA and RRB are partly covered by different items.

To test for a categorical structure of the data, latent class analyses (LCA) were performed for polytomous variables [35]. Based on literature review, we modeled LCA models with 2 to 5 classes to explore how well correlations between observed ADOS-variables can be explained by categories. LCA models were evaluated based on the Vuong-Lo-Mendell-Rubin test (LMR) [36] and BIC [37], with lower values indicating better model fit.

To test for dimensionality, a confirmatory factor analysis (CFA) was performed with categorical Maximum Likelihood Estimation as it is recommended for ordinal variables [38]. According to the model of Lord et al. [28], two factors (Social Affect (SA) and Repetitive and Restricted Behaviors (RRB)) with respective factor-item-assignment were assumed. To evaluate CFA models, we calculated the comparative fit index (CFI) [38] and root mean square error of approximation (RMSEA) [39]. Lower values of RMSEA but higher values of CFI indicate better model fit. To compare the CFA models to LCA and FMM, dimensions were additionally tested with a robust maximum likelihood estimator (ML) (= hybrid factor mixture model with one class) to compute the BIC and AIC. Additionally, the distributions of factor values were computed. In case of non-normal distribution of factor values, the model was rejected, as it is commonly assumed that the underlying dimensionality is normally distributed [40–42].

In a further step, CFA and LCA were combined into a hybrid factor mixture model (FMM) to test whether categories and dimensions are both needed to represent the structure of ASD symptoms [31]. We performed FMM with two and three classes combined with the two-factor structure (SA and RRB) with respect to the results of LCA and LMR test. FFM models were evaluated based on the LMR p values and BIC. For comparison of the different FMM models, the use of the BIC has been advocated [37], although it penalizes model complexity [43] and thus reduces the chance for hybrid models to show a low and, therefore, good BIC value compared to less complex models. Clark and colleagues [31] thus based their model selection on the BIC value, and additionally evaluated “substantive interpretation of the models using item plots” [31] (p. 9). Along this line, we complemented the BIC by an inspection and evaluation of factor value distributions, since a non-normal distribution can indicate the existence of multiple classes where there are none [41], and examined the item plots for interpretation and evaluation of the optimal model. Furthermore, the AIC was used, because it outperforms the BIC in cases of small sample sizes or difficult-to-distinguish classes [44]. Dziak et al. [43] recommend the AIC since “the most likely error in a simulation is underfitting, so the criteria with lower underfitting rates, such as AIC, often seem better. For very large *n* and easily distinguished classes, the most likely error is overfitting, so more parsimonious criteria, such as BIC, often seem better” (p. 560). Thus, we examined whether both criteria suggested the same model to best explain the data.

To allow for more appropriate comparisons of BIC, we conducted all LCA, CFA and FFM models with the Maximum Likelihood Estimation (MLR) and predefined the variables as categorical beforehand as recommended by [31, 45]. Altogether, we first identified the model with the lowest BIC and AIC. In case of CFA, we tested if factor values were normally distributed and, if not, we identified the model with the next lowest BIC and examined item plots for substantive interpretation. Additionally, to detect the correct number of latent classes we evaluated the LCA and FFM models based on the LMR test. To identify site effects within the data that are predictive of subclass membership we conducted regression analysis. As no side effects were observed, results are not further reported.

## Results

**Fig. 1:**
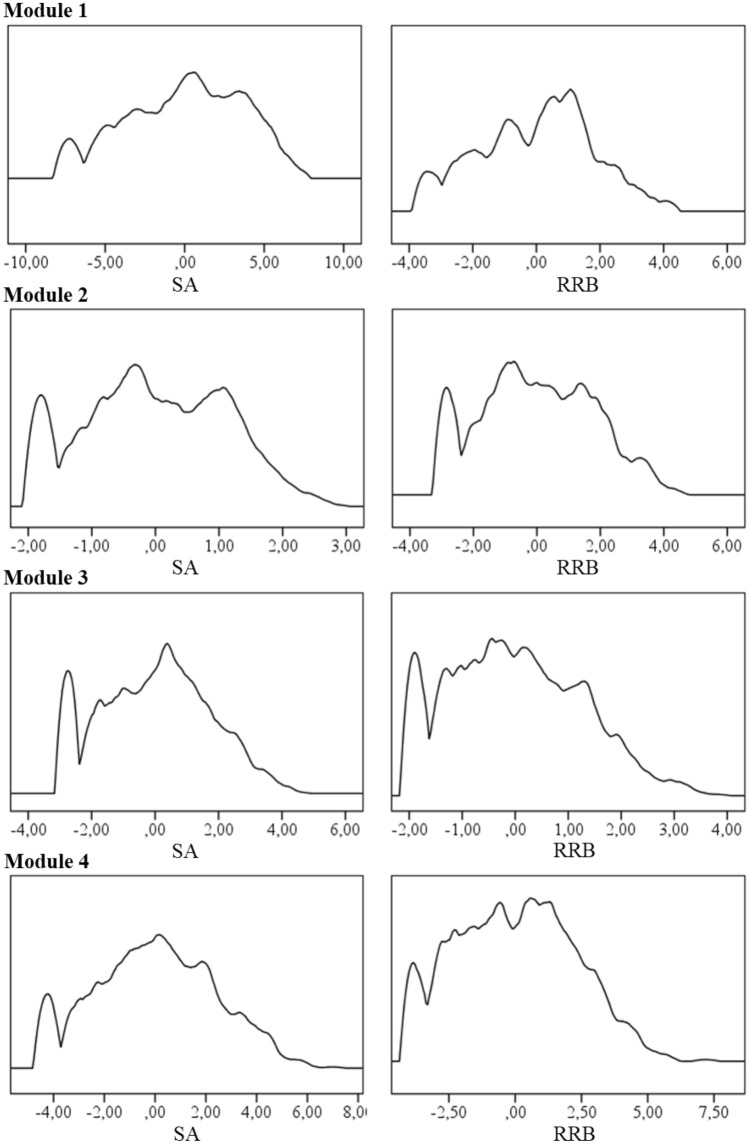
Density plots for factor distributions. Note. Density plots show the probability density of factor distribution. *SA* Social affect, *RRB* Restricted repetitive behavior

Model fit information for categorical, dimensional and hybrid models of the structure of ASD symptoms are presented in Table 2. Comparing LCA, CFA and FMM models, the dimensional two-factor CFA models show the lowest BIC value for each module (fit statistics of CFA were reported in table S2, available online). However, factor values on both factors (SA and RRB) are not normally distributed showing bi- and multimodal distributions (see density plots in Fig. 1) indicating that classes might be present going beyond the one-class dimensional CFA-based models. Additionally, Q–Q plots reject normal distributions (see Figure S1 in supplement). According to AIC values, the hybrid model with two or three classes achieves the lowest values in each module. In the following, we discuss the best overall models for each module separately.

**Table 2:** Model fit information subdivided into ADOS modules

Model	No. Classes	Log-likelihood	Parameter	BIC	LMR p-value	AIC
Module 1						
CFA		− 3863.11	37	**7951.27**		7800.23
LCA	2	− 4151.93	49	8601.89	< 0.001	8401.86
LCA	3	− 3896.69	74	8243.47	< 0.001	7941.39
LCA	4	− 3830.92	99	8263.98	0.394	7859.84
LCA	5	− 3803.75	124	8361.69	0.869	7855.50
FMM*	2	− 3830.68	62	8038.45	0.157	**7785.36**
FMM	3	− 3804.73	87	8138.61	0.787	7783.46
Module 2						
CFA		− 5569.83	43	**11,411.37**		11,225.65
LCA	2	− 5739.50	57	11,839.17	< 0.001	11,592.99
LCA	3	− 5596.37	86	11,736.16	0.761	11,364.73
LCA	4	− 5532.46	115	11,791.60	0.802	11,294.92
LCA	5	− 5477.50	144	11,864.93		11,243.00
FMM*	2	− 5479.32	72	11,413.60	0.013	**11,102.64**
FMM	3	− 5446.02	97	11,530.25	0.604	11,122.26
Module 3						
CFA		− 9213.46	42	**18,717.17**		18,510.92
LCA	2	− 9664.97	55	19,710.03	< 0.001	19,439.94
LCA	3	− 9282.92	83	19,139.44	< 0.001	18,731.85
LCA	4	− 9194.26	111	19,155.61	0.480	18,610.52
LCA	5	− 9137.26	139	19,235.12	0.147	18,552.53
FMM	2	− 9132.28	69	18,741.40	0.011	18,402.57
FMM*	3	− 9066.00	96	18,795.43	0.043	**18,324.00**
Module 4						
CFA		− 9082.62	47	**18,486.19**		18,259.24
LCA	2	− 9521.70	63	19,473.61	< 0.001	19,169.40
LCA	3	− 9165.33	95	18,979.39	0.760	18,520.66
LCA	4	− 9050.68	127	18,968.60	0.760	18,355.35
LCA	5	− 9000.74	159	19,087.25	0.760	19,319.49
FMM	2	− 9005.61	78	18,543.85	0.268	18,167.21
FMM*	3	− 8935.24	109	18,614.82	0.783	**18,088.49**

Numbers in bold font indicate the best value of all models compared with respect to BIC and AIC (lower values indicate better fit)

*CFA* Confirmatory factor analysis, *LCA* latent class analysis, *FMM* factor mixture model, *No. Classes* number of classes, *BIC* Bayesian information criterion, *AIC* Akaike information criterion

*Rows with best overall models

### Module 1

**Fig. 2:**
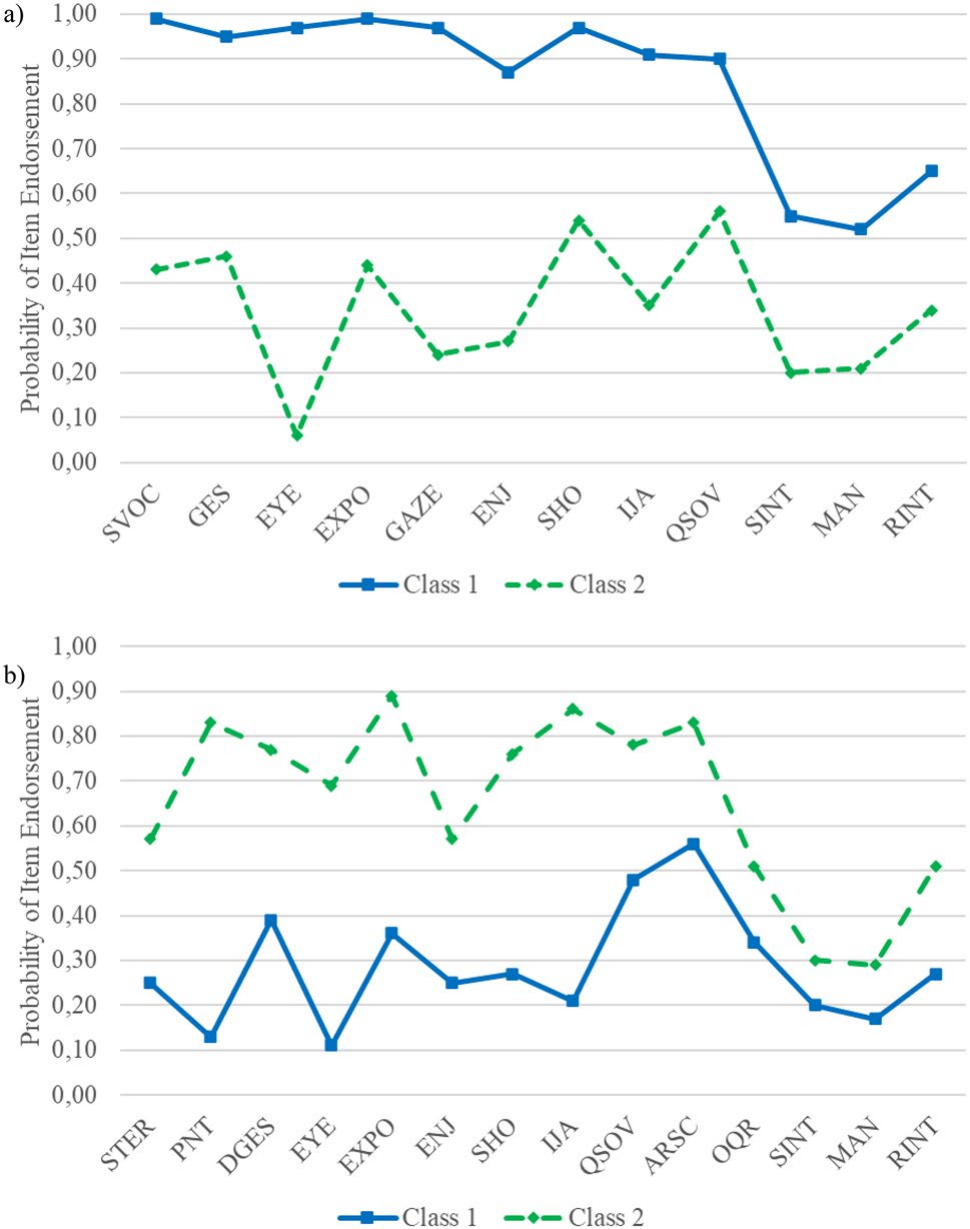
Item Profiles of the two-class FMM-Model with two Factors for ADOS module 1 **a** and module 2 **b***.* **a** Item profiles for ADOS module 1. **b** Item profiles for ADOS module 2. The *y* axis presents item endorsement probabilities for item values > 0. The *x* axis presents ADOS items (a full list of items abbreviations/ADOS keys is presented in Table S2 available online)

The model with the next lowest BIC compared to the CFA model is the two-class FMM model (BIC = 8038.45). Figure 2a demonstrates that item endorsement may be higher in class 1 (60.3% of the sample) than in class 2. For class 2, the probability of high symptom values is in a medium range, i.e., individuals of this class show lower symptom values than in class 1. This class is more likely to match the non-ASD group. In class 1, probabilities of clear evidence for abnormality according to SA lay in a high range whereas abnormalities in RRB lay in a medium range. A large proportion of this class is likely to meet criteria of an ASD diagnosis. The item plots indicate quantitative as well as qualitative differences in symptom manifestation patterns between both classes. In class 1, the probabilities of endorsement for the items “Eye Contact”, “Integration of Gaze and Other Behaviors During Social Overtures”, “Shared Enjoyment in Interaction” and “Initiation of Joint Attention” are especially low whereas endorsement probabilities for all SA items in class 2 are uniformly high. Inspection of the LMR *p*-value indicates preference for the dimensional model. The three-class FMM model has the lowest AIC value, but as the third class comprises only 4.8% of the sample, this model does not add substantial information and is thus rejected. In summary, BIC and LMR indicate preference of the dimensional model, whereas density plots (see Fig. 1), Q–Q plots (see figure S1, available online) as well as interpretability suggest a hybrid model with two classes.

### Module 2

The model with the next lowest BIC is the two-class FMM model (BIC = 11,413.60). This model has the lowest AIC value and also the LMR *p* value indicates preference for this hybrid model with two classes over the dimensional model. Class 1 (64.3% of the sample) might represent individuals with low to medium item endorsement. Probabilities of item endorsement (see Fig. 2b) are overall lower in class 1 than in class 2 with quantitative differences with regard to “Quality of Social Overtures”, “Amount of Reciprocal Social Communication” and “Overall Quality of Rapport” as well as RRB-Items. Qualitative differences in the item patterns can be observed for “Pointing”, “Showing” and “Initiation of Joint Attention” with class 2 having higher symptom endorsements. Thus, class 2 seems to represent individuals with evidence for ASD diagnoses. The three-class FMM model has the next lowest AIC value to the two-class FMM, and LMR *p* values indicate that two classes are superior to three classes. Additionally, the item plots (see Figure S2, available online) are difficult to interpret, further indicating superiority of the more conservative hybrid model with two classes.

### Module 3

**Fig. 3:**
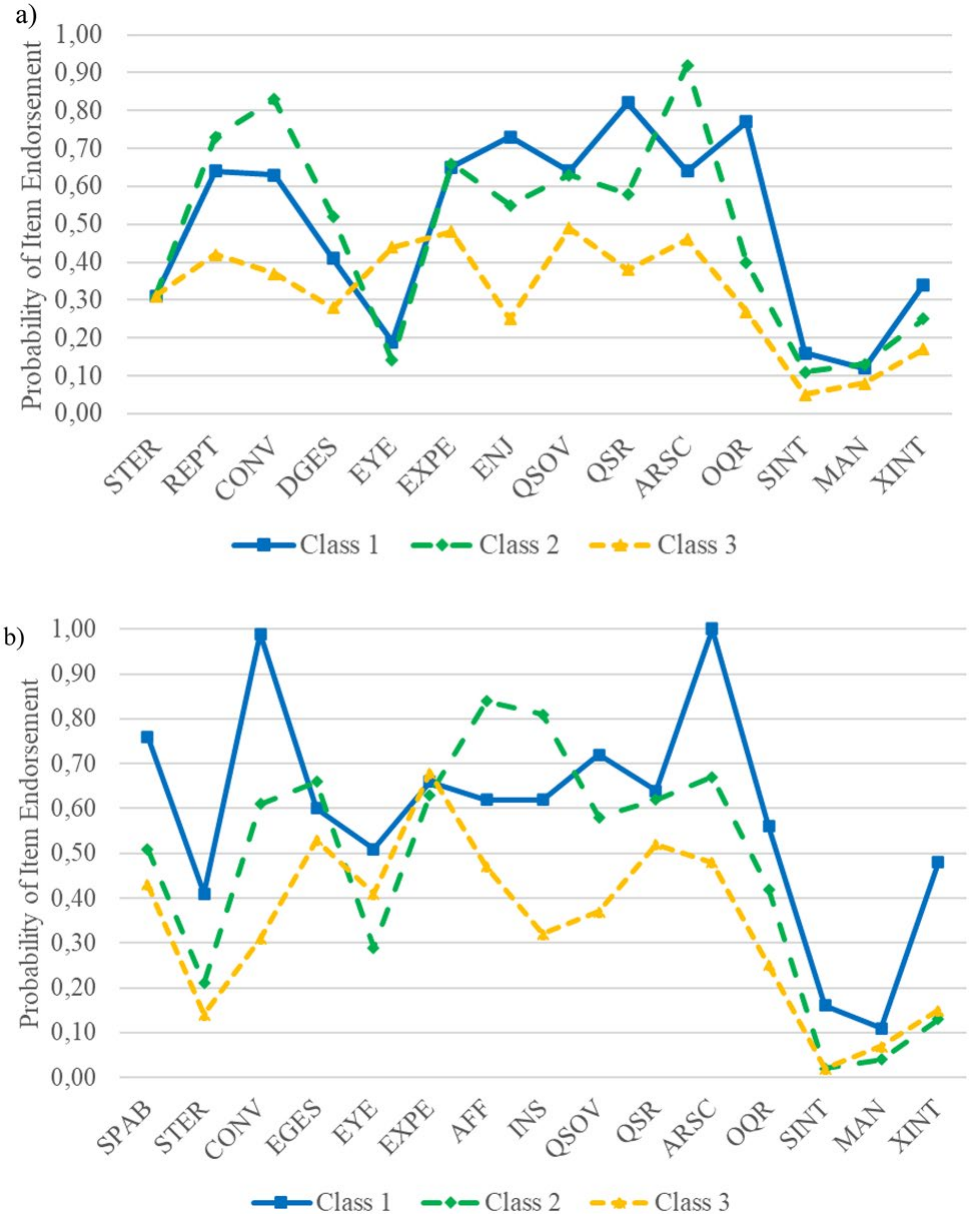
Item Profiles of the three-class FMM-3 Model with two Factors for ADOS module 3 **a** and 4 **b***.* **a** Item profiles for ADOS module 3. **b** Item profiles for ADOS module 4. The *y* axis presents item endorsement probabilities for item values > 0. The *x* axis presents ADOS items (a full list of items abbreviations/ADOS keys is presented in Table S2 available online)

The two-class FMM model (BIC = 18,741.41) is the model with the next lowest value after the CFA model and, according to the LMR test, the hybrid model with two classes is superior to CFA. Inspection of model parameters did not lead to good interpretability, because the probabilities of item endorsement are quite similar (see Figure S3, available online). Thus, AIC and LMR test suggest the FMM three-class solution (BIC = 18,795.43). Class 3 (51.3% of the sample) has low to medium probabilities for endorsement of items (see Fig. 3a). A comparison of the classes’ item endorsement profiles shows lower values in class 3 compared to classes 1 and 2 and differences in profiles except for “Eye-Contact”. Thus, class 3 seems to represent the asymptomatic class (non-ASD). Class 1 (23.1%) scores higher on the items “Shared Enjoyment”, “Quality of Social Response” and “Overall Quality of Rapport”. Class 2 (25.5%) scores higher on the items “Conversation” and “Amount of Reciprocal Social Communication”. Probabilities for endorsement of all three items of the RRB domain and “Reporting of Events” are similar. Thus, individuals in class 1 may have higher symptom scores than those in class 2 and may meet criteria for ASD diagnosis (ASD subgroup A). Class 2 might represent individuals with symptom scores in a medium range (ASD subgroup B). In summary, results for Module 3 indicate that the hybrid model with three classes fits best.

### Module 4

The two-class FMM model has the lowest BIC with 18,543.85 next to the CFA model, but the LMR test suggests preference for the dimensional model. Class 1 (37.1% of the sample) shows qualitative differences to class 2 due to a different profile of item endorsement probability. However, some items differ only slightly between these classes (see figure S4, available online). The model with the next lowest BIC and the lowest AIC is the FMM model with three classes (class 1 = 9.9% of the sample, class 2 = 47.5%, class 3 = 42.6%). Individuals in class 3 show low to medium item endorsements (see Fig. 3b) and might comprise asymptomatic individuals (non-ASD). Classes 1 and 2 differ concerning the domain Social Affect. Class 1 is the highly endorsed symptom class, with particularly high probabilities for endorsement of the items “Conversation” and “Amount of Reciprocal Social Communication” (see Fig. 3b). This class might represent individuals meeting the diagnostic criteria for ASD (ASD subgroup A). Class 2 might show endorsed symptoms but in a lower range than class 1 (ASD subgroup B), except for “Communicating of Affect” and “Insight”. In summary, considering BIC and AIC values, interpretability and LMR tests, we found mixed evidence for a hybrid model to explain behavioral data in adolescents and adults.

### Overall

Taking BIC, AIC, value distribution, LMR test and examination of item pattern plots for substantive interpretation into account when comparing LCA, CFA and FMM models, we found mixed results. We did not find conclusive evidence for a dimensional distribution of ASD symptoms. Instead, our results hint at a hybrid latent structure. Beyond evaluation criteria, our results suggest that the percentage of individuals with high probabilities for item endorsement in the FFM models match the amount of ASD cases in the respective ADOS module (for example: module 1 included 58.7% cases with a diagnosis of ASD and in the class with high symptom profiles were 60.3% of all cases; see Table 3).

**Table 3:** Proportion of the sample with and without a diagnosis of autism across the classes

Module	ASD diagnoses (%)	Class with evidence for ASD (%) in FFM model
1	58.7	60.3
2	39.5	35.7
3	44.3	48.6
4	54.4	57.7

Subtype-classes A and B in module 3 and 4 were added up

## Discussion

A fundamental, yet unresolved, question is whether ASD is a qualitatively distinct condition (= categorial), has a dimensional latent structure along a continuum of severity, or whether it is best described as a hybrid model combining categorical and dimensional attributes. In the present study, considering the BIC value, the dimensional models demonstrated the best fit suggesting a dimensional latent structure of the ASD symptoms. Evaluation of factor values, however, showed multimodal distributions leading to the rejection of entirely dimensional models. Furthermore, AIC values indicated a consistent preference of the hybrid models. Therefore, models with the next lowest BIC values and with the lowest AIC were evaluated regarding their implications about the latent structure of ASD symptoms. Based on our results combined with clinical considerations, we conclude that the hybrid models best represent the latent structure of ASD behavior symptoms.

For Modules 1, the FMM model achieves good interpretability with regard to what is observed in clinical practice. One group of patients shows abnormalities with some symptom overlap to ASD (= class 2), whereas another group shows significant behavioral problems according to SA and RRB, especially in those symptoms which were found to be predictive for a diagnosis of ASD (e.g. “Eye Contact”, “Integration of Gaze and Other Behaviors During Social Overtures”, “Initiation of Joint Attention” (for review see [46])). In Module 2, we also found two classes, but with more similarities and some probability for item endorsement. In class 2, we found higher probabilities for endorsement of items from the SA and RRB domains. Thus, there is evidence for hybrid models with two classes (ASD and non-ASD) and two factors (SA and RRB) which is in line with the DSM-5 proposed hybrid approach and with findings by Frazier et al. [11]. Again, we found qualitative differences in the most consistent features of young children with ASD: Behaviors associated with joint attention—which is considered to be one of the first steps of a cascade leading to social cognition deficits [47]. Altogether, our non-ASD sample consists of individuals drawn from a clinical sample including other mental and developmental disorders sharing symptoms with ASD. Qualitative differences in younger children (Module 1 and 2) might by minimal but nevertheless meaningful.

The hybrid models for Modules 3 and 4 suggest three classes (non-ASD, ASD subgroup A and ASD subgroup B) and two factors (SA and RRB). This is in contrast to Frazier and colleagues [11] who also found a three-class model—comparing children with ASD with their mostly healthy siblings—but argued that this model did not add substantial information regarding different subgroups of ASD (e.g., autism, Asperger). The three classes can be described as follows: one class with a minimal to low symptom profile and two classes with mild to high symptom profiles that differ in their pattern. In Module 4, the ASD subgroup A class shows item endorsement for a broad range of items. The ASD subgroup B shows probabilities for general endorsement in a medium range and high peaks for the items “Conversation” and “Amount of Reciprocal Social Communication”. This profile may represent individuals with a Social Communication Disorder according to DSM-5, as their impairment occurs mainly in the domain of communication and severity in the domain of RRB is low (with the exception of “Excessive Interest”). However, this interpretation has to be taken with caution as it is based solely on behavioral observation data. Interestingly, whereas in younger children the item “Eye Contact” clearly differs between the classes, in verbally fluent children, adolescents and adults it does not, but instead, differences in verbal aspects do.

Altogether, we found evidence for at least two classes: One non-ASD class and one to two ASD subgroup-classes that are qualitatively different from each other due to characteristic patterns of symptoms. Additionally, it has to be taken into account that we examined difficult-to-distinguish classes (ASD and a non-ASD group with symptom overlap with ASD). This aspect is also visible in Fig. 3a, 3b as there is no class with low probability for all ADOS items. Nevertheless, we found evidence for clinically relevant differences between the groups. These differences are more visible in the younger (Modules 1 and 2) than the older sample (module 3 and 4). This corresponds with clinically results that higher functioning individuals present milder symptoms and usually come to clinical presentation later in life [48] and individuals with other mental disorder also present symptoms of ASD or autistic traits [49–51].

Whereas we found evidence for the hybrid model with two classes for the younger children with no fluent speech (module 1 and 2), we found three classes for the older children, adolescents and adults (module 3 and 4). This could indicate that the developmental course of ASD results in the differentiation of subgroups of ASD. However, this suggestion is thill to be investigated by longitudinal studied. On the other hand, our results could imply that the subgroups are due to age, cognitive and/or language level of participants or based on differential severity level as found by Georgiades et al. [22]. The finding of subgroups of ASD is in line with many other studies (e.g. [22]) finding two to four subtypes (see [52] for review), but will have to be validated in independent samples and follow-up data. Nonetheless, this result is highly relevant as the identification of different subtypes has important implications for both, the understanding of etiology and interventions for ASD.

### Clinical implications

Despite the results of empirical analyses [53] and the insights regarding the best criteria for the decision on the best fitting model [43], many clinicians and researchers advocate the use of a dimensional approach to conceptualize ASD. A continuum is assumed with the endpoints of “autism” versus “neurotypical”. Thereby, “autism” is described as a continuous dimension and an “ego syntonic” label that comprises the personality (“autistic”) and is defined in particular by its strengths resulting in high identification and less stigma [54–56]. The problem, however, is the lack of specificity of single ASD symptoms and of current diagnostic criteria in the distinction to many other mental disorders. This results in broadening the concept of ASD and, consequently, in substantial heterogeneity of the ASD phenotype, complicating the investigation of the (biological) basis for the condition. Therefore, effect sizes from cognitive, EEG and neuroanatomical studies comparing ASD and control samples have dropped by 80% over time [57] and the eightfold increase of studies investigating “autistic traits” in the general (“neurotypical”) population and in other clinical conditions [23]. A central focus for future research of ASD is, therefore, to understand the heterogeneity [58] and the identification of distinguishable subgroups of ASD [59].

In clinical contexts, dimensional approaches are usually translated back into categorical approaches via the use of cut-off points to determine the degree of severity necessary for a “formal” diagnosis. In most countries/jurisdictions, a “formal” diagnosis is necessary for treatment over and above the question whether the patient is eligible for certain programs or therapies. As there is no objective marker and “the dimensional conception of autism has no natural cut-off point where high autism traits become ‘autism” [60] (p. 228), the diagnostic decision is built on subjective judgements or personal experience of the patient (whether he/she feels to be “on the spectrum”) as well as the examiner [61]. Especially, as concepts such as “masking”, “compensation”, and “camouflaging” [62, 63] have become increasingly popular, the value of standardized behavioral observations [28] and interviews with caregivers [64] has decreased, the criterion of an early onset of ASD has been undermined and heterogeneity has increased. Like a vicious circle, the hope of finding valid biomarkers is additionally hampered by the dramatic increase of heterogeneity and comorbidity in ASD. Currently, we note a trend towards the inclusion of individuals with autistic traits as a basis for research on autism whereas considerations of differential diagnoses are rare. Thus, we believe there is a danger of losing touch with what is called “prototypical autism” [19].

Altogether, we conclude that within our current knowledge, the hybrid model is the best fitting model for ASD. This conclusion is also in line with our as well as several other studies using different methodological designs or biological markers [8–10, 65, 66] and seems most reasonable from a clinical perspective in terms of diagnostic usefulness. Additionally, in the context of public health decisions (e.g., access to service provision), a hybrid approach is sensible and necessary, as it is both grounded in empirical evidence and in clinical usefulness. The hybrid approach allows for the classification of individuals into diagnostic groups, relevant subgroups and at the same time accounts for within—class differences in severity. It is in line with the current classification systems and has the potential of being more specific, decreasing false positive diagnoses. Our results underline the urgent need for research in subtypes of ASD [17]. Furthermore, as the symptom profiles of the different classes demonstrate overlapping but also different profiles, the hybrid model appears more suitable for the early detection of ASD cases and may help to differentiate ASD from other mental disorders compared to a purely dimensional approach. Taking the symptom profiles into account could for example improve diagnostic accuracy [67].

### Strengths and limitations

A main strength, in contrast to previous studies [5, 11, 23], is the well characterized and balanced sample of (unrelated) ASD cases and individuals with highly relevant differential diagnoses which were all evaluated with “gold standard” assessment tools for ASD. We did not include a typically developing, healthy sample. As we analyzed the different ADOS modules separately, one limitation of our study is that the sample sizes are small in contrast to the samples of other studies. Although we are correcting for several biases based on parental information, we cannot rule out examiner bias. Furthermore, our results are solely based on the ADOS items [68]. Whether the psychopathology of one individual is describable and/or understandable through a categorical and/or dimensional label seems highly questionable. Many other aspects (course, comorbidity, age at diagnosis, core and peripheral symptoms) may play important roles and could influence the probability of item endorsement, resulting in different classes in taxonomic analyses. Future studies should include measures of further psychopathology or functioning (e.g., negative and positive valence, adaptive functions). Another limitation is, that we only tested a two-factor solution for the ADOS, but there is some evidence for higher dimensional solutions (subdimensions in the domain of Social affect; see [69, 70]) or a bifactor structure [68]. Additionally, due to the non-normal distribution of symptoms, alternative modeling approaches such as zero-inflated models, unipolar models should to be considered (see [42]) to examine a potential dimensional structure that assumes a non-normal latent variable without distinct classes. To fully understand the heterogeneity of ASD and whether there are distinguishable subgroups is another important research question that could not being addressed sufficiently in this study. Future studies should consider these possibilities.

### Supplementary Information

Below is the link to the electronic supplementary material.

Supplementary file1 (DOCX 319 KB)
