# Supplementary material for: Conceptualization of the latent structure of autism: further evidence and discussion of dimensional and hybrid models
Source: Eur Child Adolesc Psychiatry. 2022 Aug 25;32(11):2247–58. doi: 10.1007/s00787-022-02062-y (PMC10576682; doi:10.1007/s00787-022-02062-y)
Supplement: Supplementary file 1 — Supplementary file1 (DOCX 319 KB) [file 787_2022_2062_MOESM1_ESM.docx]

**Table S1**

*Frequencies of Main Disorders Included into the Sample*

| Disorder | Frequency (%) |
| --- | --- |
| Autism spectrum disorder | 1424 (48.8) |
| Without axis one diagnosis | 334 (11.4) |
| Substance use disorder (F10-F19) | 11 (0.4) |
| Schizophrenia, schizotypal and delusional disorders (F20-F29) | 9 (0.3) |
| Mood disorders (F30-F39) | 86 (2.9) |
| Neurotic, stress-related and somatoform disorders (F40-F48) | 98 (3.4) |
| Behavioural syndromes associated with physiological disturbances and physical factors (F50-F59) | 2 (0.1) |
| Disorders of adult personality and behaviour (F60-F69) | 78 (2.7) |
| Mental Retardation (F70- F79) | 81 (2.77) |
| Disorders of psychological development (other than ASD) (F80-F89) | 335 (11.4) |
| Behavioural and emotional disorders with onset usually occurring in childhood and adolescence (F90-F98) | 462 (15.8) |

*Note*. *N* = 2920.

**Table S2**

*Items and Items Abbreviations of all ADOS Modules*

| Key | Variable Name |
| --- | --- |
| Social Affect Dimension | |
| AFF | Communication of Own Affect |
| ARSC | Amount of Reciprocal Social Communication |
| ASOV | Amount of Social Overtures / Maintenance of Attention |
| CONV | Conversation |
| DGES | Descriptive, Conventional, Instrumental, or Informational Gestures |
| EGES | Emphatic or Emotional Gestures |
| ENJ | Shared Enjoyment in Interaction |
| EXPE/EXPO | Facial Expressions Directed to Examiner/Other |
| EYE | Unusual Eye Contact |
| GAZE | Integration of Gaze and Other Behaviors During Social Overtures |
| INJ | Initiation of Jount Attention |
| INS | Insight |
| OQR | Overall Quality of Rapport |
| QSOV | Quality of Social Overtures |
| QSR | Quality of Social Response |
| Restricted, repetitive Behavior Dimension | |
| MAN | Hand and Finger and Other Complex Mannerisms |
| SINT | Unusual Sensory Interest in Play Material/Person |
| SPAB | Speech Abnormalities Associated With Autism |
| STER | Stereotyped/Idiosyncratic Use Of Words or Phrases |
| XINT | Excessive Interest in or References to Unusual or Highly Specific Topics or Objects or Repetitive Behaviors |

*Note*. This is a list of items abbreviations of all ADOS Modules, but not all items are included in all modules of the ADOS, therefore the items in this table and those in Figures 2-4 can differ.

**Table S3**

*Fit Statistics for Factor Analyses*

| Module No. | χ² | df | p-value | CFI | TLI | RMSEA | SRMR |
| --- | --- | --- | --- | --- | --- | --- | --- |
| 1 | 85.20 | 53 | .003 | .998 | .997 | .037 | .028 |
| 2 | 260.08 | 76 | <.001 | .974 | .969 | .066 | .054 |
| 3 | 334.62 | 76 | <.001 | .980 | .977 | .058 | .046 |
| 4 | 423.54 | 89 | <.001 | .972 | .967 | .064 | .056 |

*Note*. df = Degrees of freedom. CFI = Comparative Fit Index. TLI = Tucker-Lewis Index. RMSEA = Root Mean Square Error of Approximation. SRMR = Standardized Root Mean Square Residual. Higher values of CFI and TLI indicate better fit. Lower values of RMSEA and SRMR indicate better fit.

**Table S4**

*Factor Values and Item Thresholds for two-class FMM of Module 1*

|  |  | | Item threshold | | | | | | | |  |
| --- | --- | --- | --- | --- | --- | --- | --- | --- | --- | --- | --- |
|  | | | class 1 | | | | class 2 | | | |  |
|  | Factor loadings | 1 | | 2 | | 1 | | 2 | |  |  |
| Social Affect | | | | | | | | | | | |
| SVOC | 1.00 | | -6.41 | | -1.84 | | 0.46 | | 3.25 | |  |
| GES | 0.47 | | -3.31 | | -0.84 | | 0.19 | | 1.79 | |  |
| EYE | 0.52 | |  | | -4.02 | |  | | 3.63 | |  |
| EXPO | 1.13 | | -6.85 | | -1.23 | | 0.43 | | 31.81 | |  |
| GAZE | 0.99 | | -5.19 | | -1.37 | | 1.96 | | 4.09 | |  |
| ENJ | 0.62 | | -2.44 | | -0.08 | | 1.32 | | 4.03 | |  |
| SHO | 0.81 | | -4.81 | | -1.99 | | -0.27 | | 1.32 | |  |
| IJA | 0.92 | | -3.58 | | -1.51 | | 1.02 | | 2.67 | |  |
| QSOV | 1.11 | | -6.86 | | -0.5 | | -0.43 | | 3.82 | |  |
| Restricted repetitive Behavior | | | | | | | | | | | |
| SINT | 1.00 | | -0.37 | | 1.90 | | 2.28 | | 4.16 | |  |
| MAN | 0.43 | | -0.11 | | 11.2 | | 1.53 | | 2.71 | |  |
| RINT | 0.50 | | -0.74 | | 1.01 | | 0.82 | | 3.42 | |  |

*Note*. A full list of item abbreviations/ADOS keys is presented in Table S2. Social Affect Factor: Factor mean = 0.00, standardized factor variance = 1.14. Restricted repetitive Behavior Factor: Factor mean = 0.00, standardized factor variance = 1.95.

Item thresholds reported for each class for item values 1 and 2.

**Table S6**

*Factor Values and Item Thresholds for three-class FMM of Module 3*

|  |  | Item threshold | | | | |  |
| --- | --- | --- | --- | --- | --- | --- | --- |
|  |  | class 1 | | | class 2 | |  |
|  | Factor loading | | 1 | 2 | 1 | 2 |  |
| Social Affect | | | | | | | |
| PNT | 1.00 | -1.63 | | 2.00 | 1.99 | 4.05 |  |
| DGES | 2.71 | -1.54 | | 0.46 | 0.57 | 2.02 |  |
| EYE | 2.94 |  | | -1.00 |  | 2.62 |  |
| EXPO | 4.90 | -3.38 | | 1.98 | 0.99 | 5.58 |  |
| ENJ | 5.22 | -0.50 | | 2.78 | 1.93 | 4.4 |  |
| SHO | 2.28 | -1.39 | | 0.72 | 1.17 | 2.76 |  |
| IJA | 2.04 | -2.04 | | 0.95 | 1.54 | 2.52 |  |
| QSOV | 7.74 | -2.98 | | 4.26 | 0.19 | 6.74 |  |
| ARSC | 6.87 | -3.28 | | 0.42 | -0.52 | 3.32 |  |
| OQR | 5.03 | -0.06 | | 3.71 | 1.17 | 4.47 |  |
| Restricted repetitive Behavior | | | | | | | |
| STER | 1.00 | -0.44 | | 2.22 | 1.64 | 4.04 |  |
| SINT | 0.72 | 1.08 | | 3.14 | 1.77 | 4.49 |  |
| MAN | 0.51 | 1.00 | | 2.30 | 1.79 | 2.95 |  |
| RINT | 1.01 | -0.05 | | 2.88 | 1.45 | 4.21 |  |

*Note*. A full list of item abbreviations/ADOS keys is presented in Table S2. Social Affect Factor: Factor mean = 0.00, standardized factor variance = 0.46. Restricted repetitive Behavior Factor: Factor mean = 0.00, standardized factor variance = 0.58.

Item thresholds reported for each class for item values 1 and 2.

**Table S7**

*Factor Values and Item Thresholds for three-class FMM of Module 4*

|  |  | Item threshold | | | | |  |
| --- | --- | --- | --- | --- | --- | --- | --- |
|  |  | class 1 | | | class 2 | |  |
|  | Factor loading | | 1 | 2 | 1 | 2 |  |
| Social Affect | | | | | | | |
| CONV | 1.00 | -7.63 | | 1.41 | -0.88 | 2.42 |  |
| EGES | 0.68 | -0.61 | | 2.60 | -0.99 | 0.52 |  |
| EYE | 0.76 |  | | -0.05 |  | 3.72 |  |
| EXPE | 1.16 | -1.33 | | 9.76 | -1.09 | 3.72 |  |
| AFF | 0.51 | -0.65 | | 1.55 | -2.13 | 1.21 |  |
| INS | 0.74 | -0.79 | | 1.21 | -2.22 | 0.73 |  |
| QSOV | 1.03 | -1.75 | | 3.72 | -0.64 | 2.81 |  |
| QSR | 1.00 | -1.11 | | 4.25 | -0.90 | 4.52 |  |
| ARSC | 1.50 | -13.00 | | 4.30 | -1.83 | 2.60 |  |
| OQR | 0.69 | -0.34 | | 1.85 | 0.45 | 2.60 |  |
| Restricted repetitive Behavior | | | | | | | |
| SPAB | 1.00 | -2.18 | | 2.63 | -0.05 | 4.14 |  |
| STER | 0.37 | 0.44 | | 2.65 | 1.58 | 4.97 |  |
| SINT | 0.44 | 2.05 | | 13.07 | 4.28 | 5.56 |  |
| MAN | 0.31 | 2.29 | | 3.97 | 3.43 | 4.82 |  |
| XINT | 0.43 | 0.09 | | 2.69 | 2.29 | 4.28 |  |

*Note*. A full list of item abbreviations/ADOS keys is presented in Table S2. Social Affect Factor: Factor mean = 0.00, standardized factor variance = 1.06. Restricted repetitive Behavior Factor: Factor mean = 0.00, standardized factor variance = 1.69.

Item thresholds reported for each class for item values 1 and 2.

**Table S6**

*Factor Values and Item Thresholds for three-class FMM of Module 3*

|  | | Item threshold | | | | | |
| --- | --- | --- | --- | --- | --- | --- | --- |
|  |  | class 1 | | class 2 | | class 3 | |
|  | Loading | 1 | 2 | 1 | 2 | 1 | 2 |
| Social Affect | | | | | | | |
| REPT | 1.00 | -0.86 | 1.06 | -1.43 | 0.9 | 0.49 | 3.24 |
| CONV | 1.65 | -1.09 | 2.24 | -3.06 | 1.56 | 1.08 | 4.76 |
| DGES | 0.96 | 0.54 | 1.77 | -0.11 | 3.64 | 1.25 | 3.26 |
| EYE | 1.73 |  | 2.92 |  | 3.49 |  | 0.52 |
| EXPE | 1.45 | -1.1 | 4.50 | -1.19 | 4.89 | 0.15 | 3.84 |
| ENJ | 1.22 | -1.63 | 1.69 | -0.33 | 3.02 | 1.77 | 4.44 |
| QSOV | 1.71 | -1.17 | 3.02 | -1.06 | 3.93 | 0.08 | 4.20 |
| QSR | 1.48 | -2.71 | 3.08 | -0.56 | 6.88 | 0.93 | 5.81 |
| ARSC | 2.29 | -1.45 | 3.46 | -5.59 | 1.84 | 0.39 | 5.01 |
| OQR | 1.27 | -1.99 | 2.04 | 0.7 | 30.7 | 1.63 | 5.38 |
| Restricted repetitive Behavior | | |  |  |  |  |  |
| STER | 1.00 | 1.24 | 4.43 | 1.24 | 5.63 | 1.24 | 4.67 |
| SINT | 0.72 | 2.18 | 5.80 | 2.75 | 4.9 | 3.63 | 6.40 |
| MAN | 0.88 | 2.76 | 4.48 | 2.73 | 4.31 | 3.41 | 4.74 |
| XINT | 0.55 | 0.84 | 3.26 | 1.32 | 3.68 | 1.89 | 4.07 |

*Note*. A full list of item abbreviations/ADOS keys is presented in Table S2. Social Affect Factor: Factor mean = 0.00, standardized factor variance = 0.26. Restricted repetitive Behavior Factor: Factor mean = 0.00, standardized factor variance = 0.84.

Item thresholds reported for each class for item values 1 and 2.

**Figure S1**

Q-Q-Plots

**
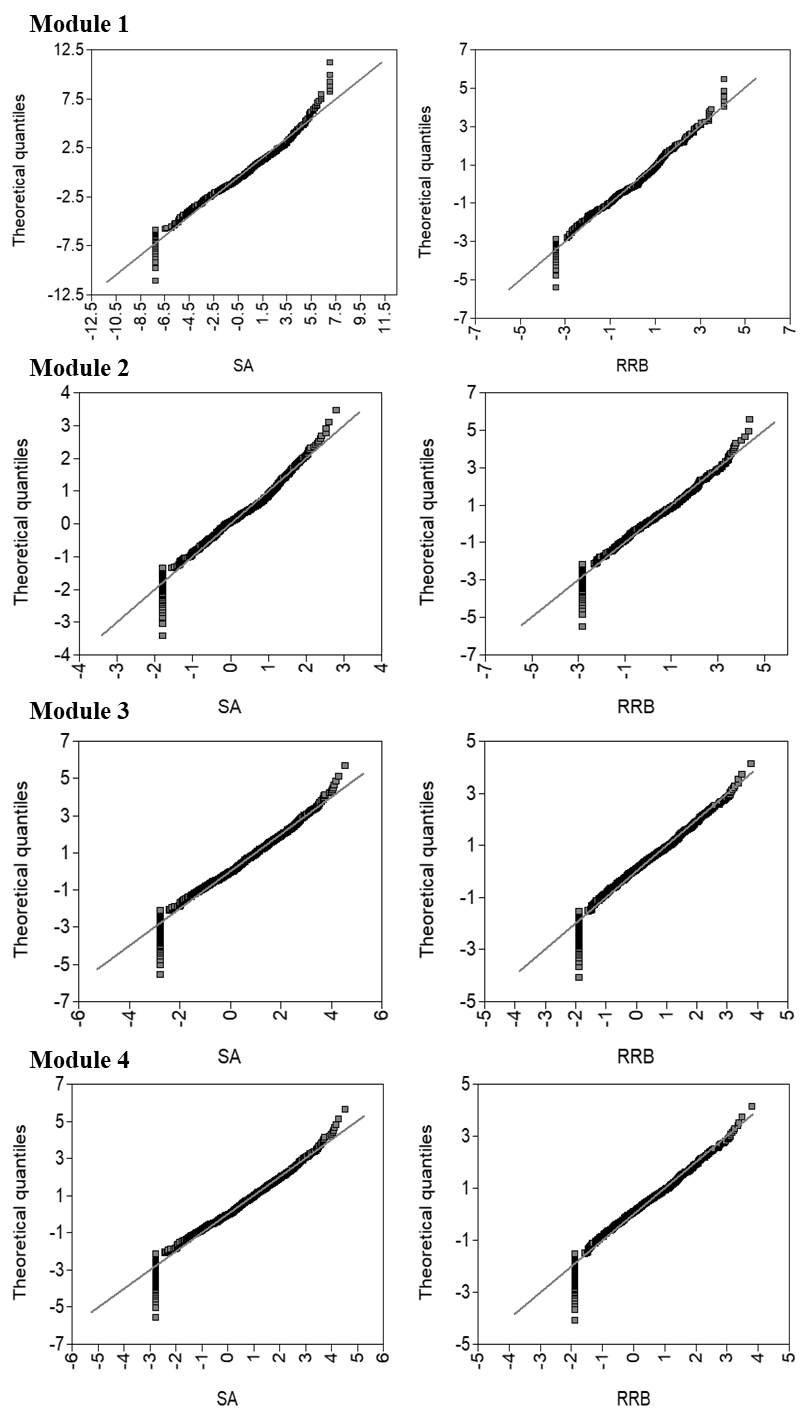
**

*Note.* Q-Q-plot allows for comparison between the observed factor values (SA and RRB) and the expected normal distribution (theoretical quantiles). A greater deviation of the dots from the diagonal indicated greater deviation of factor values from a normal distribution. SA = Social Affect. RRB = Restricted repetitive Behavior.

**Figure S2**

*Item Profiles of the 3-class FMM-Model of Module 2*


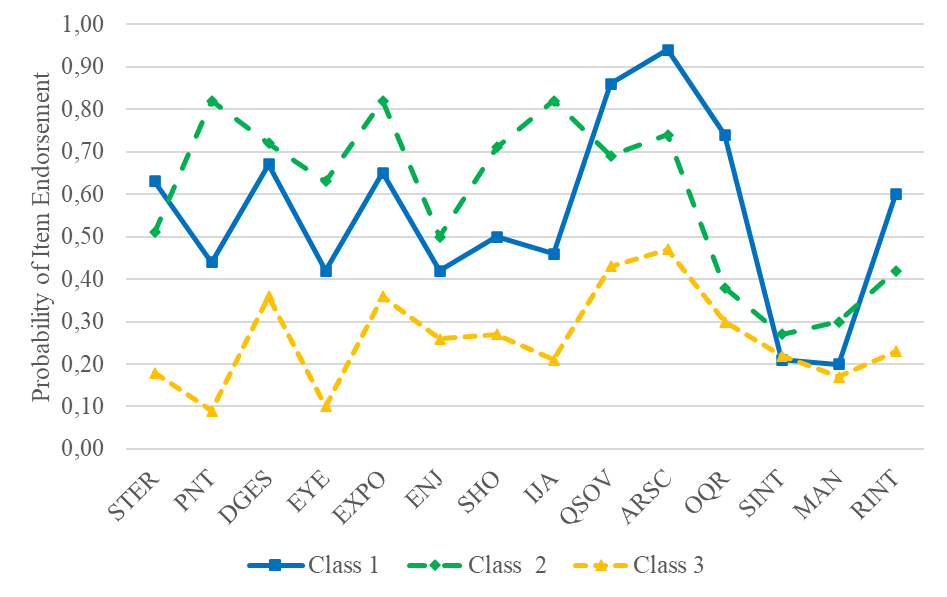


*Note.* Item endorsement probabilities for item values > 0 are presented on the y-axis. ADOS items are presented on the x-axis (a full list of item abbreviations/ADOS keys is presented in Table S2).

**Figure S3**

*Item Profiles of the 2-class FMM-3 of Module 3*
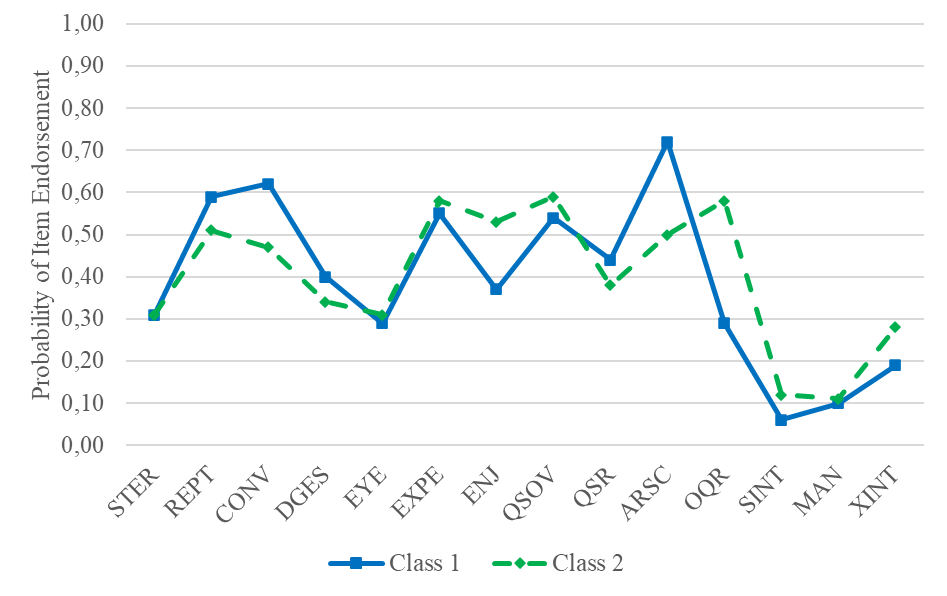
*Note.* Item endorsement probabilities for item values > 0 are presented on the y-axis. ADOS items are presented on the x-axis (a full list of item abbreviations/ADOS keys is presented in Table S2).

**Figure S4**

*Item Profiles of the 2-class FMM-Model of Module 4*


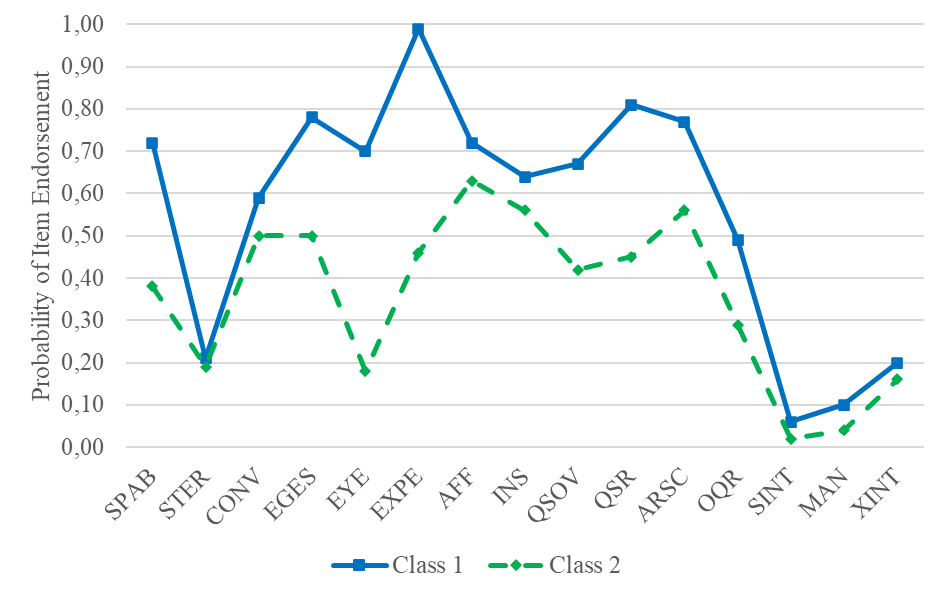


*Note.* Item endorsement probabilities for item values > 0 are presented on the y-axis. ADOS items are presented on the x-axis (a full list of item abbreviations/ADOS keys is presented in Table S2).
